# Preliminary evidence of altered neural response during intertemporal choice of losses in adult attention-deficit hyperactivity disorder

**DOI:** 10.1038/s41598-018-24944-5

**Published:** 2018-04-30

**Authors:** Saori C. Tanaka, Noriaki Yahata, Ayako Todokoro, Yuki Kawakubo, Yukiko Kano, Yukika Nishimura, Ayaka Ishii-Takahashi, Fumio Ohtake, Kiyoto Kasai

**Affiliations:** 1 ATR Brain Information Communication Research Laboratory Group, 2-2-2 Hikaridai, Seika-cho, Soraku-gun, Kyoto, 619-0288 Japan; 2 Department of Neuropsychiatry, Graduate School of Medicine, The University of Tokyo, 7-3-1 Hongo, Bunkyo-ku, Tokyo, 113-8655 Japan; 3 Department of Child Neuropsychiatry, Graduate School of Medicine, The University of Tokyo, 7-3-1 Hongo, Bunkyo-ku, Tokyo, 113-8655 Japan; 4 Global Center of Excellence Program ‘Comprehensive Center of Education and Research for Chemical Biology of the Diseases’, The University of Tokyo, 7-3-1 Hongo, Bunkyo-ku, Tokyo, 113-8655 Japan; 5 Department of Molecular Imaging and Theranostics, National Institute of Radiological Sciences, National Institutes for Quantum and Radiological Science and Technology, 4-9-1 Anagawa, Inage, Chiba, 263-8555 Japan; 6 Graduate School of Economics, Osaka University, 1-7 Machikaneyamacho, Toyonaka, Osaka, 560-0043 Japan; 7 The International Research Center for Neurointelligence (WPI-IRCN) at The University of Tokyo Institutes for Advanced Study (UTIAS), The University of Tokyo, 7-3-1 Hongo, Bunkyo-ku, Tokyo 113-8655 Japan

## Abstract

Impulsive behaviours are common symptoms of attention-deficit hyperactivity disorder (ADHD). Although previous studies have suggested functional models of impulsive behaviour, a full explanation of impulsivity in ADHD remains elusive. To investigate the detailed mechanisms behind impulsive behaviour in ADHD, we applied an economic intertemporal choice task involving gains and losses to adults with ADHD and healthy controls and measured brain activity by functional magnetic resonance imaging. In the intertemporal choice of future gains, we observed no behavioural or neural difference between the two groups. In the intertemporal choice of future losses, adults with ADHD exhibited higher discount rates than the control participants. Furthermore, a comparison of brain activity representing the sensitivity of future loss in the two groups revealed significantly lower activity in the striatum and higher activity in the amygdala in adults with ADHD than in controls. Our preliminary findings suggest that an altered size sensitivity to future loss is involved in apparent impulsive choice behaviour in adults with ADHD and shed light on the multifaceted impulsivity underlying ADHD.

## Introduction

Attention-deficit hyperactivity disorder (ADHD) is characterized by inattention, impulsivity, and hyperactivity and affects 3.4% of children^1^, with approximately 65% of children with ADHD continuing to have some symptoms in adulthood^2^. One indication of impulsive behaviour is ‘impulsive choice’, that is, choosing a smaller but immediate reward over a larger but delayed reward from the intertemporal choices presented. The intertemporal choice paradigm has been widely used to evaluate an individual’s temporal preference in economic surveys^3–8^ and neuroimaging studies^9–20^, as well as in clinical studies involving individuals with ADHD^21–30^. A recent meta-analysis of studies on impulsive choice in individuals with ADHD provided robust evidence that choice behaviour is more impulsive in individuals with ADHD than in control participants^31^. Some studies on impulsive choice in ADHD have indicated that individuals with ADHD show a strong negative affect when delay is imposed and, in light of this, ‘choosing immediate gains to avoid the slightest delay’ is the cause of the impulsive choice observed in ADHD^24–27,32^.

The delay aversion model, however, is concerned with gains alone and omits the factor of losses. Previous findings indicated that individuals with ADHD exhibit a dysfunctional response to losses. For example, children with ADHD require a larger response cost (monetary loss) to inhibit their reactions in the Go/no-go task or Stop task compared with control children^33–35^. A clinical study found that children with ADHD are prone to drug, alcohol, and gambling addictions as adults^36^. Another model of impulsive choice in ADHD that does take into account the specific features of both gains and losses^37,38^ proposes that individuals with ADHD cannot resist the temptation of immediate gains because of a reduced sensitivity to future negative outcomes caused by choosing immediate gains. This means that not only delay aversion to gains, but also reduced sensitivity to future negative outcomes, can cause impulsive choice behaviour.

Previous studies have shown differences in brain activity between adults with ADHD and healthy adults. For example, adults with ADHD show abnormalities in the corticostriatal loops, which are involved in delay discounting^39^, and in the dopamine system, leading to aberrant sensitivity to the size of both gains and losses^40^. Given these previous findings on brain function, we hypothesized that the brain areas (including the striatum) receiving projections from dopaminergic neurons, as well as the frontal areas (prefrontal, orbitofrontal, and medial prefrontal cortices, and insula) that have connections to the striatum, are involved in the delay discounting of gains and losses and that the brain activity associated with size sensitivity in these areas shows a difference specifically in loss prediction between ADHD and healthy individuals.

A recent study suggested the importance of investigating delay discounting in adults with ADHD^41^. Although ADHD has traditionally been considered a disorder of childhood and impulsive choice has been reported in children and adolescents with ADHD^42^, adults diagnosed as having ADHD in childhood report difficulties in social and economic activities, such as higher school dropout rates, underemployment, compromised job performance, difficulty maintaining employment, and problems maintaining friendships^43^. This suggests that investigation of impulsive choice in adults with ADHD is needed to understand fully the mechanism of impulsive behaviour in ADHD.

Thus, to understand the mechanism of the impulsive behaviour observed in ADHD, it is necessary to evaluate the effects of both ‘delay’ and ‘gain/loss’ factors. In our previous study, we developed an intertemporal choice task that can estimate an individual’s temporal preference for both gains and losses^10^. Here, by measuring brain activity using functional magnetic resonance imaging (fMRI) during an intertemporal choice task with gain/loss, we investigated the effects of the ‘delay’ and ‘gain/loss’ factors on behavioural and neural levels in patients with ADHD.

## Results

### Behavioural results

**Figure 1:**
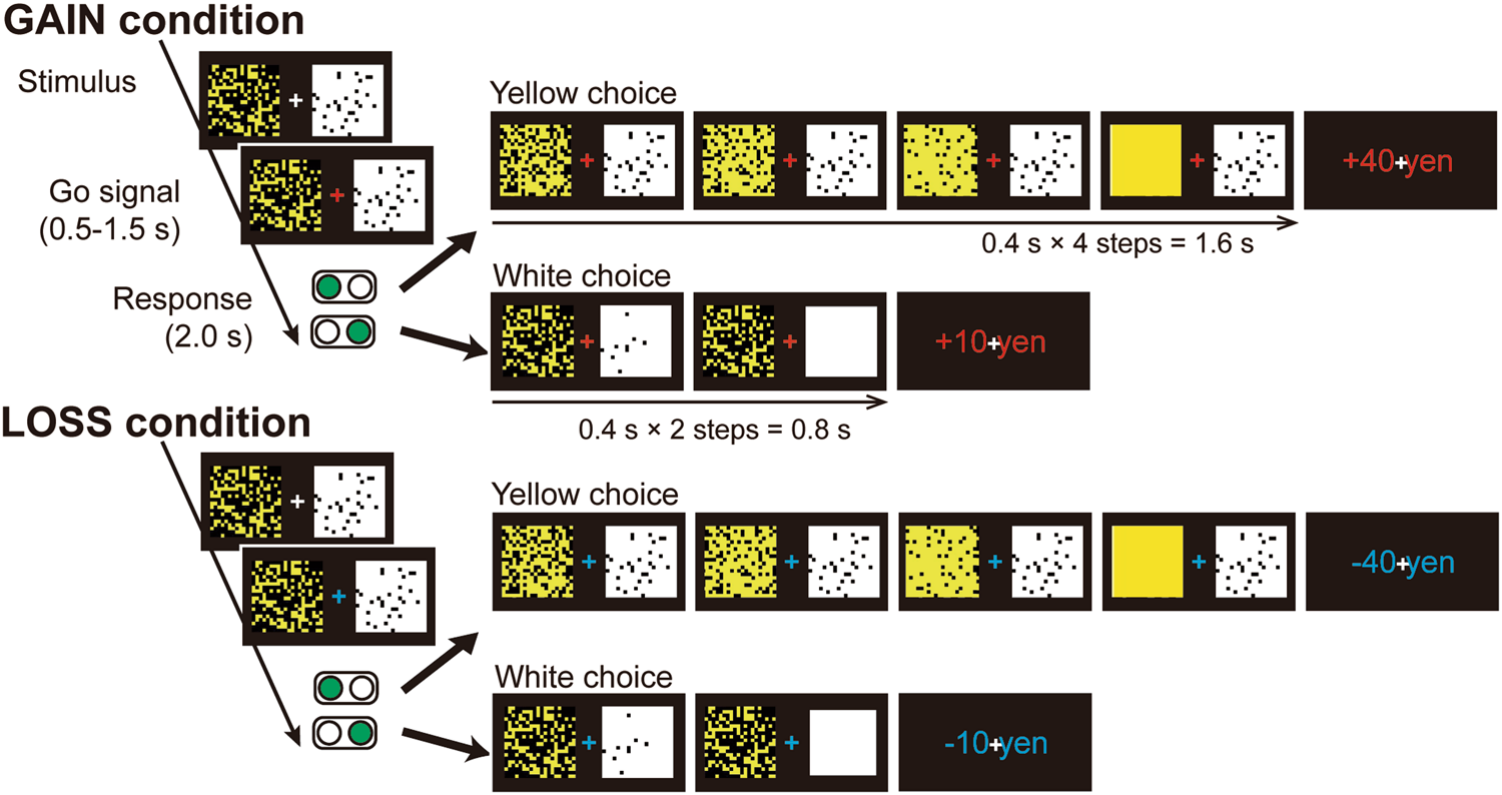
Participants were asked to select either a white or yellow square by pressing a button on the corresponding side. In the example shown, if the participant chose a white square at each step, a small monetary gain (10 yen) in the GAIN condition or a small monetary loss (−10 yen) in the LOSS condition was delivered in two steps (0.4 × 2 = 0.8 s). If the participant chose a yellow square, four yellow choices (0.4 × 4 = 1.6 s) had to be repeated to obtain a larger monetary gain (40 yen) in the GAIN condition or a larger monetary loss (−40 yen) in the LOSS condition. The position of the squares (left or right) was randomly changed at each trial.

Fifteen adults with ADHD and 19 normal control (NC) participants (see Table 1 for participants’ demographic characteristics) performed repeated intertemporal choice tasks^10^ for future gains (GAIN condition) and losses (LOSS condition) (Fig. 1; see Methods), selecting either a white or yellow square corresponding to a smaller (+10/−10 yen) or larger (+40/−40 yen) reward/punishment option, respectively. We estimated the inverse discount rate of each participant based on his or her individual choice pattern in the GAIN and LOSS conditions.

**Table 1:** Demographic characteristics of the participants.

	ADHD (*N* = 15)		Normal controls (*N* = 19)		Group difference (*P*-value)
	Mean	s.d.	Mean	s.d.	
Sex (M/F)	5/10		7/12		0.84
Age (years)	31.33	6.5	32.42	6.0	0.62
Education (years)	15.4	2.4	16.9	1.5	0.031
JART	109.3	9.6	113.3	6.4	0.15
WAIS-R or III	110.0	17.7	—	—	—
ASRS part A (0–6)	4.9	0.8	0.95	0.85	<0.01
ASRS part B (0–12)	6.3	1.9	1.3	1.1	<0.01
WURS (0–100)	64.6	17.0	19.9	11.5	<0.01
GAF (1–100)	69.1	13.4	85.2	8.7	<0.01

M, male; F, female; JART, Japanese version of the National Adult Reading Test; WAIS-R or III, Wechsler Adult Intelligence Scale–Revised or 3rd Edition; ASRS, Adult ADHD Self-Report Scale; WURS, Wender Utah Rating Scale; GAF, Global Assessment of Functioning.

In economic theory, intertemporal choice is explained by the delay discounting model^44^; the value of a delayed outcome is discounted temporally, *discounted value* = *u*(*outcome)* × *g*(*delay*), where *u* is the utility function and *g* is the discount function, which decreases with an increasing length of the delay. The ‘indifference point’ method was used to calculated each participant’s degree of discounting based on his or her individual choice patterns^45^. The delay length of the larger-later option (*D*_*L*_) was plotted against the delay length of the smaller-sooner option (*D*_*S*_) for each choice. Logistic regression analysis was used to fit the probability of choosing the larger-later option (*P*_*L*_) as follows^9,11^:

1 $${P}_{L}=1/(1+{\rm{e}}{\rm{x}}{\rm{p}}[-({\beta }_{L}{D}_{L}+{\beta }_{S}{D}_{S}+{\beta }_{0})]).$$

when *P*_*L*_ = 0.5, this equation is transformed to

2 $${D}_{L}=-\,{\beta }_{{S}}/{\beta }_{{L}}{D}_{{S}}-{\beta }_{{0}}/{\beta }_{{L}}.$$

**Figure 2:**
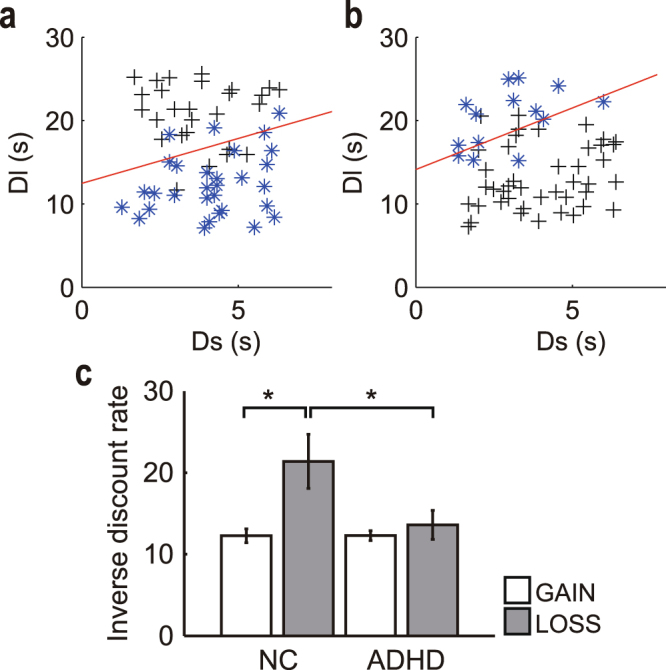
(**a**) Choice pattern of a representative participant (NC) in the GAIN condition and (**b**) the LOSS condition. The delay length of the larger-later option (D_L_) was plotted against the delay length of the smaller-sooner option (D_S_) for each choice. Asterisks (*) indicate selection of the larger-later option (yellow square: +40 yen) and crosses (+) indicate selection of the smaller-sooner option (white square: +10 yen). The lines indicate the estimated indifference line of the participant [intercept = 12.47 (GAIN), 14.13 (LOSS) for the representative participant]. The intercept of the indifference line inversely reflects the discount rate. **(c)** Estimated inverse discount rate. In the LOSS condition, there was a significantly smaller discount rate in NCs than in adults with ADHD (*P < 0.05, multiple comparison with the Tukey–Kramer method). The NCs showed a significantly smaller discount rate in the LOSS condition (grey bars) than in the GAIN condition (white bars) (*P < 0.05, Tukey–Kramer method). All error bars denote ± 1 s.e.m. (*n* = 19 for NCs, *n* = 15 for ADHD).

Logistic regression analysis provides the coefficients (*β*_*L*_, *β*_*S*_, and *β*_0_). Figure 2a and b show examples of indifference lines in the GAIN and LOSS conditions, respectively. For any point on this ‘indifference line’, the discounted values of the smaller-sooner and larger-later options were equal.

By applying the method of indifference points to the specific discount model, we can compute an individual discount rate from the estimated intercept. The theoretical model of hyperbolic discounting *g*(*D*) = 1/(1 + *k*_*h*_*D*) can be transformed to the same form as the indifference line equation in the following way:

3 $$\begin{array}{c}1/(1+{k}_{h}{D}_{L})\times u(40)=1/(1+{k}_{h}{D}_{S})\times u(10)\\ {D}_{L}=b{D}_{S}+c/{k}_{h}\,{\rm{in}}\,{\rm{the}}\,{\rm{hyperbolic}}\,{\rm{model}},\\ b=u(40)/u(10),c=(u(40)-u(10))/u(10)\end{array}$$

Here, *u*(40) and *u*(10) are the utility for 40 yen and 10 yen, respectively. We adopted a linear utility function here; thus, *u*(40) = 40 and *u*(10) = 10. *k*_*h*_ is the discount rate and is included in the intercept. The intercept in Equation (2) −*β*_0_/*β*_*L*_ reflects the ‘inverse discount rate’ *c*/*k*_*h*_ in Equation (3).

Figure 2c shows the inverse discount rates of adults with ADHD and NCs in the GAIN and LOSS conditions (NC/GAIN: mean = 12.50, s.d. = 3.74, *n* = 19; NC/LOSS: mean = 21.81, s.d. = 14.21, *n* = 19; ADHD/GAIN: mean = 11.54, s.d. = 2.61, *n* = 15; ADHD/LOSS: mean = 12.99, s.d. = 6.36, *n* = 15). A 2 × 2 mixed analysis of variance (ANOVA) was used to analyse the inverse discount rates for gains and losses, with condition (GAIN/LOSS) as the within-subject factor and group (NC/ADHD) as the between-subject factor. A significant interaction (*F*_1,32_ = 4.75, *P* = 0.037, *η*_*p*_^*2*^ = 0.129) and a significant condition effect (*F*_1,32_ = 8.91, *P* = 0.005, *η*_*p*_^*2*^ = 0.218) were found. Multiple comparison analysis of inverse discount rates revealed a significant difference between the two groups in the LOSS condition; a smaller inverse discount rate (i.e., more impulsivity) was found in adults with ADHD than in the NCs (*P* < 0.05, Tukey–Kramer method). In the GAIN condition, no significant difference was found between the groups (*P* > 0.1, Tukey–Kramer method). These results suggest that adults with ADHD show a smaller discounted value for future losses than healthy adults, even though their choice behaviour stays the same in intertemporal choice tasks involving gains.

Multiple comparison analysis also revealed a significant difference between the GAIN and LOSS conditions in NCs (*P* < 0.05, Tukey–Kramer method): NCs showed a larger inverse discount rate (i.e., less impulsivity) in the LOSS condition than in the GAIN condition. No such significant difference was seen in adults with ADHD (*P* > 0.1, Tukey–Kramer method). This result suggests that NCs exhibited asymmetry in the discounting of gains and losses, whereas adults with ADHD did not.

### Imaging results

**Figure 3:**
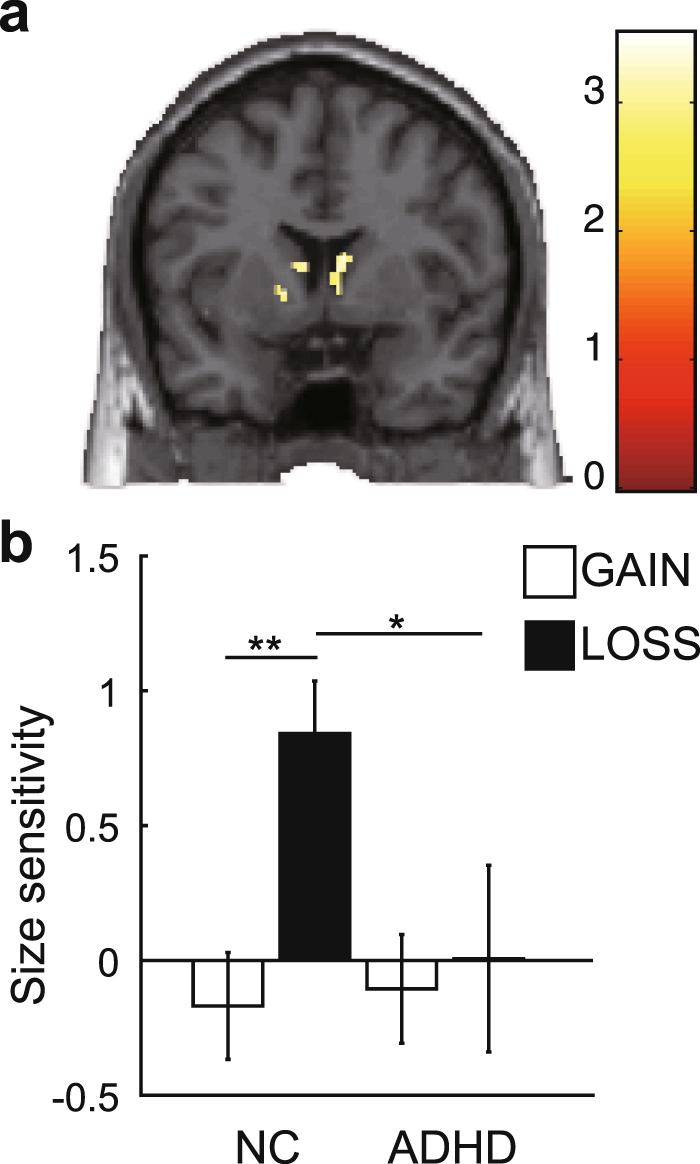
(**a)** Result of a group comparison (NCs > ADHD) of the BOLD signal for size sensitivity in the LOSS condition (see Supplementary Table S1; *P* < 0.05 for visualization). (**b**) BOLD signal for size sensitivity in the GAIN (white bars) and LOSS (grey bars) conditions in the anatomically defined region of interest of the left and right caudate heads (red coloured area) (*P < 0.05, **P < 0.001, multiple comparison with the Tukey–Kramer method). Error bars denote ±1 s.e.m. (*n* = 17 for NCs, *n* = 14 for ADHD).

We evaluated the effect of condition (gain/loss) at the neural level. We subtracted event-related blood-oxygen-level dependent (BOLD) signals for choosing smaller-sooner options from event-related BOLD signals for choosing larger-later options, where a small difference between the BOLD signals would indicate weak gain/loss size sensitivity at the neural level. First, we simply performed a group comparison using a two-sample t-test in the GAIN and LOSS condition separately. In the LOSS condition, we found a significantly lower activation in adults with ADHD than in NCs in the caudate (*P* < 0.05, corrected; Fig. 3 and Supplementary Table S1). In the GAIN condition, on the other hand, no significant difference was observed (with *P* < 0.01, uncorrected). In the BOLD signal representing size sensitivity in the caudate, we observed a significant condition effect (Fig. 3b; *F*_1,29_ = 4.98, *P* = 0.033, *η*_*p*_^*2*^ = 0.147; NC/GAIN: mean = −0.17, s.d. = 0.82, *n* = 17; NC/LOSS: mean = 0.84, s.d. = 0.80, *n* = 17; ADHD/GAIN: mean = −0.11, s.d. = 0.75, *n* = 14; ADHD/LOSS: mean = −0.0067, s.d. = 1.29, *n* = 14). On multiple comparison, BOLD signals representing size sensitivity were significantly weaker in adults with ADHD than in NCs in the LOSS condition only (Fig. 3b; *P* < 0.05, Tukey–Kramer method). These results suggest that neural activity representing size sensitivity is reduced in adults with ADHD and that this reduction occurs only in the LOSS condition. We also found significantly greater activity representing size sensitivity in the LOSS condition than in the GAIN condition in NCs alone (Fig. 3b; *P* < 0.001, Tukey–Kramer method). This result suggests that the NCs exhibited asymmetry in the size sensitivity in gains and losses, whereas adults with ADHD did not, as observed in discounting behaviour.

**Figure 4:**
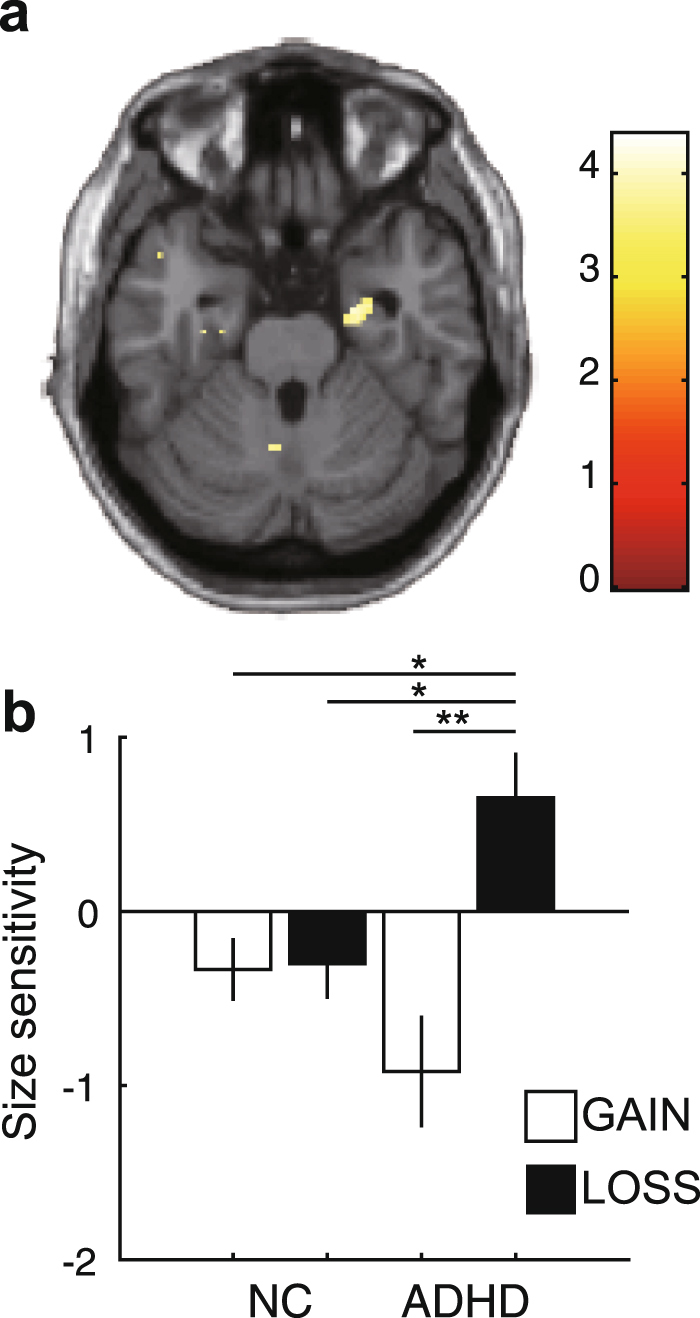
(**a**) Result of the interaction with group and condition (gain/loss) of the BOLD signal for size sensitivity (see Supplementary Table S2; *P* < 0.05 for visualization). (**b**) BOLD signal for size sensitivity in the GAIN (white bars) and LOSS (grey bars) conditions in the anatomically defined region of interest of the left and right caudate heads (red coloured area) (*P < 0.01, **P < 0.001, multiple comparison with the Tukey–Kramer method). Error bars denote ± 1 s.e.m. (*n* = 17 for NCs, *n* = 14 for ADHD).

Next, to evaluate an interactive effect between gain/loss and group, we employed a factorial analysis with condition and group factors. A significant interaction was found in temporal areas, including the amygdala (*P* < 0.05, small-volume corrected; Fig. 4a and Supplementary Table S2). On multiple comparison, BOLD signals representing size sensitivity in the amygdala were significantly greater in adults with ADHD than in NCs in the LOSS condition (Fig. 4b; *P* < 0.01, Tukey–Kramer method; NC/GAIN: mean = −0.33, s.d. = 0.74, *n* = 17; NC/LOSS: mean = −0.30, s.d. = 0.83, *n* = 17; ADHD/GAIN: mean = −0.92, s.d. = 1.20, *n* = 14; ADHD/LOSS: mean = 0.65, s.d. = 0.97, *n* = 14). This result was the opposite of the behavioural and activity in the caudate.

We also evaluated the effect of delay at the neural level. We defined the correlation between the event-related BOLD signals and the delay length of the chosen options as delay-related activity. A group comparison using a two-sample t-test for the GAIN and LOSS conditions separately found no significant difference between ADHD and NCs (*P* > 0.001, uncorrected). Factorial analysis revealed no significant interaction (*P* > 0.001, uncorrected). This suggests that brain activity representing delay length did not differ significantly between the two groups in either the GAIN or LOSS condition.

## Discussion

At the behavioural level, we found that adults with ADHD discounted future losses as much as future gains, although the control participants discounted future losses less than future gains. Moreover, we found a significant difference between the groups in discounting for losses, not for gains. This lack of a difference in discounting for gains is consistent with previous studies that used intertemporal choice tasks in which the total length of experimental time was not reduced when a smaller immediate gain was chosen^21–23^. However, contradictory findings have demonstrated that adults with ADHD exhibit impulsive choice in intertemporal choice tasks with gains^24–27,29,30^. One reason may be that impulsivity in temporal discounting is mostly observed in children and adolescents with ADHD^25,27,29,30^. Only adults with ADHD participated in this study, which may be why we did not find impulsive choice in the gain condition in ADHD. We confirmed that there was no significant difference in age between the ADHD and NC groups (Table 1) and also that there was no significant relationship between age and discounting parameters (the inverse discount rates) in both the ADHD and NC groups. To examine the effect of age—children versus adults—on discounting with gains and losses, we should apply our experimental protocols to children with ADHD. Another reason is that most previous studies have presented the intertemporal choice task as a questionnaire. In doing so, the time scale often ranges from days to months; in the present study, we used time scales ranging from several seconds to several tens of seconds. To evaluate to what extent adult participants and different time scales of delay might affect the sensitivity of the task, future studies should clarify the effect of the time scale on the temporal discounting of both gains and losses for both children and adults with ADHD.

At the neural level, we obtained data indicating striatal insensitivity to loss in our adult participants with ADHD. Many previous studies have demonstrated that specific brain areas, such as the prefrontal cortex, insula, and striatum, are involved in temporal discounting of future appetitive outcomes, such as drinks and monetary rewards^9,12–20^. In the striatum in particular, some previous studies reported a positive relationship between the striatal BOLD response and monetary loss^46,47^. We observed a significant difference between the groups in the activity related to loss size sensitivity in the striatum. Aberrant sensitivity to the size of outcomes has also been reported in ADHD studies^48,49^, and individuals with ADHD have been shown to have little or no change in skin conductance in response to negative outcomes compared with healthy normal individuals^50–52^. A previous study proposed that corticostriatal circuits are dysfunctional in ADHD^53^; therefore, it is possible that the striatum is involved in loss discounting and, because the striatal circuit is dysfunctional in ADHD, individuals with ADHD have an impaired response to future losses in intertemporal choice problems. Indeed, adults with ADHD showed abnormalities in the corticostriatal loops, which are involved in delay discounting^39^, and in the dopamine system, leading to aberrant sensitivity to the size of both gains and losses^40^. This hypothesis may be viewed as a loss version of the functional model of ADHD proposed by Sonuga-Barke and Fairchild^53^, in which individuals with ADHD exhibit diminished responses to gains because of a dysfunctional orbitofrontal cortex–striatum circuit (or dopaminergic circuitry).

A recent meta-analysis revealed that the striatum, including the nucleus accumbens and caudate, is involved in delay discounting^54^. We performed ROI analysis of the nucleus accumbens (Supplementary Fig. S1). Although we did not find a main effect of the condition (*P* = 0.063) and interaction (*P* > 0.1) in the size sensitivity of the nucleus accumbens, the activation pattern was similar to that of the caudate. We found a significant difference between the GAIN and LOSS conditions in NCs (*P* = 0.0055), but not in the participants with ADHD (*P* > 0.1). This result also supports our finding that ADHD patients did not exhibit the sign effect in the neural level in the caudate and nucleus accumbens. However, we did not observe a significant difference between groups in the LOSS condition (*P* > 0.1). This result suggests that the caudate is more sensitive to the LOSS in NCs than the nucleus accumbens and that a significant difference between groups in the LOSS was observed.

Our result suggests a difference in the involvement of the amygdala in loss discounting between adults with ADHD and healthy participants, with ADHD individuals hypersensitive to the loss. The amygdala has been suggested as one of the important loci in ADHD^55^. Previous studies found amygdala hyperactivation in ADHD during emotional processing of negative outcomes, including anticipation and experience of monetary loss^56,57^. De Martino *et al*. demonstrated that individuals with amygdala lesions exhibit a distinct reduced sensitivity to monetary losses compared with matched controls. On the other hand, hyperactivation of the amygdala has been reported in delayed outcome processing in adults with ADHD^23,58–60^, suggesting a link between a neural alteration in the amygdala in delayed outcome processing and behavioural delay aversion in ADHD. Based on these previous findings, we suggest that our finding of hyperactivation of the amygdala in loss size sensitivity in ADHD is involved not only in loss processing, but also in delay processing in our loss discounting task. Interestingly, we found the opposite activation pattern of loss size sensitivity in the caudate and amygdala, namely, hypoactivation and hyperactivation in adults with ADHD. This different involvement of the caudate and amygdala in loss discounting in ADHD should be further examined.

Here, the control participants discounted future losses less than future gains. In behavioural economics, this observed asymmetry in discounting between gains and losses is called the ‘sign effect’^3–5^. Our finding is that adults with ADHD, in contrast to the controls, did not exhibit the sign effect. Also, loss size insensitivity in the caudate in our adult participants with ADHD indicates that they had weaker ‘loss aversion’^6^ compared with healthy adults, because loss aversion is suggested to be the main cause of the sign effect^7^. Along these lines, because healthy individuals show loss aversion, future losses are predicted to be larger than the actual losses, which lowers apparent discount rates and inhibits impulsive behaviour. This would mean that our adult participants with ADHD did not predict future losses to be larger than the actual losses because of weaker loss aversion.

In the theoretical model of temporal discounting, not only ‘reduced loss size sensitivity’, but also ‘enhanced loss delay sensitivity’ may be causal factors for the sign effect or seemingly high discount rates for losses. If a future loss is perceived as a more distant event than it is in actuality, its discounted value at the time of decision-making becomes smaller, leading to the larger apparent discount rate. The analytical method used in this study to reveal the brain activity associated with size sensitivity and delay allowed us to investigate the two causal factors separately for seemingly high discount rates for future losses. Indeed, in our previous study, we observed ‘reduced loss size sensitivity’ in the insula and ‘enhanced loss delay sensitivity’ in the striatum in the individuals who did not exhibit the sign effect^10^. The present results showed no difference in delay-related activity in the loss condition, thereby eliminating the possibility of ‘enhanced loss delay sensitivity’ and allowing us to identify ‘reduced loss size sensitivity’ in the ADHD group. However, because we used only two different sizes in each condition, we could evaluate only one type of size sensitivity in this study. To understand to what extent size sensitivity is reduced or whether the absolute reward values influence the intensity of size sensitivity, it is necessary to use an experimental paradigm that allows for assessment of size-related and delay-related systems separately by manipulating size and delay independently, as seen in studies using rats^61^.

In conclusion, we used a neuroeconomics approach with an intertemporal choice task for gains and losses to demonstrate that altered loss size sensitivity results in apparent impulsive choice behaviour in adults with ADHD. Our findings shed light on the multifaceted impulsivity underlying ADHD. A limitation of this study was the modest sample size; thus, larger studies are required to evaluate the present findings and reveal the detailed mechanism of impulsive behaviour in ADHD and other psychiatric disorders.

## Methods

### Participants

Twenty-two Japanese right-handed adults with ADHD were recruited from the outpatient clinic at the Department of Neuropsychiatry, The University of Tokyo Hospital, Japan. The diagnostic procedure included an approximately 2-h interview based on the Diagnostic and Statistical Manual of Mental Disorders, Fourth Edition (DSM-IV)^62^ criteria and the Mini-International Neuropsychiatric Interview (MINI)^63^ for comorbid psychiatric conditions by an experienced child psychiatrist (A.T. or A.I.T.; clinical experience >7 years). The diagnosis of ADHD was confirmed through subsequent visits to the outpatient clinic, with supplementary use of information from parents, school report cards (assessments and comments by teachers regarding the child’s behaviour at school), and data from the Adult ADHD Self-Report Scale (ASRS)^64^, Wender Utah Rating Scale (WURS)^65,66^, and AQ. The final consensus diagnosis was made through a clinical conference among child psychiatrists and psychologists (A.T., A.I.T., H.K., Y.Kan., and Y.Kaw.). Subsequently, Conners’ Adult ADHD Diagnostic Interview for DSM-IV™ (CAADID) and Conners’ Adult ADHD Rating Scale (CAARS) became available in Japan in 2012 and were applied to 8 of the 14 participants, confirming the diagnosis of ADHD.

The presence of psychopathological co-morbidity was screened using MINI and six patients had dysthymia, three had generalized anxiety disorder, and one had obsessive-compulsive disorder [one was suspected to have alcohol dependence upon screening with the MINI, but this was later ruled out by a trained psychiatrist (A.T.) upon interview]. The following exclusion criteria were applied: (1) comorbid current major depressive disorder; (2) history of manic episode; (3) schizophrenia; (4) any sign of neurological disorder; (5) history of electroconvulsive therapy; (6) alcohol or substance abuse or dependence as defined by DSM-IV; (7) use of a psychotropic drug within 4 weeks of MRI data acquisition, and (8) a full-scale IQ < 85 on the Wechsler Adult Intelligence Scale–Revised (WAIS-R)^67^ or 3rd Edition (WAIS-III)^68^. We also excluded one participant with ADHD due to a young age (16 years old).

Twenty-two Japanese age-, sex-, dominant handed-, and IQ-matched healthy NCs participated as the control group. The presence of ADHD symptoms and other psychopathological symptoms and diseases was evaluated in a manner similar to the patient group evaluation. The NCs were interviewed by a trained psychiatrist (A.T.) to screen for neuropsychiatric disorders. The exclusion criteria applied to the control group were: (1) ADHD symptom scale scores exceeding the cutoff value; (2) neurological illness; (3) traumatic brain injury with any known cognitive consequences or loss of consciousness for more than 5 min; (4) alcohol/substance abuse or addiction; and (5) history of psychiatric disease. We also excluded one NC who chose only the larger-later option in the GAIN condition because we could not estimate any discount parameter.

We excluded one adult with ADHD and two NCs from the fMRI data analysis because there was a problem with the synchronization between the fMRI scanner and the PC running the experimental task. As a result, behavioural data were acquired from 15 medication-naïve adults with ADHD [5 men and 10 women; mean age 31.3 (s.d. = 6.5) years] and 19 NC [7 men and 12 women; mean age 32.4 (s.d. = 6.0) years] and imaging data were acquired from 14 medication-naïve adults with ADHD [5 men and 9 women; mean age 31.2 (s.d. = 6.7) years] and 17 NC [6 men and 11 women; mean age 33.0 (s.d. = 5.7) years].

All participants were right-handed, as determined by the Edinburgh Inventory^69^ with a laterality index of 0.8 as the cutoff for right-handedness. IQ was assessed with the full-scale WAIS-R or III for the adults with ADHD and with the Japanese version of the National Adult Reading Test^46^ for the NCs. Level of social functioning was evaluated using the Global Assessment of Functioning (GAF)^62^. The demographic characteristics of the participants are shown in Table 1. Because we recruited IQ-matched healthy NCs, the ADHD group exhibited a shorter duration of education than the control group. If we were to try to match the duration of education between the NC and ADHD groups, only high-functioning patients would be recruited. To avoid such a bias, we prioritized IQ over duration of education.

The Ethics Committee of the Faculty of Medicine, The University of Tokyo, approved this study (No. 3048). After receiving a complete explanation of the study, all participants provided written informed consent. All methods were carried out in accordance with the approved guidelines and regulations.

### Experimental task

We used a repeated intertemporal choice with monetary gains and losses^10^. In economic studies, the ‘intertemporal choice task’ has been commonly used to estimate a participant’s discount rate for both gains and losses^3–8^. In an economic intertemporal choice task, the participant chooses a smaller-sooner monetary gain/loss or a larger-later monetary gain/loss in the form of a questionnaire. A typical questionnaire contains several choices with a fixed delay length and different monetary amounts. To measure neural activity when computing the discounted value, we conducted a repeated intertemporal choice task^11^ with monetary gains and losses^10^. In the repeated intertemporal choice task, which we have described previously^10^, the participants chose between a smaller-sooner and a larger-later option in each trial. At the start of each trial, a white square (smaller-sooner option) and a yellow square (larger-later option), occluded by a varying number of black patches indicating the monetary payoff delays, were shown side-by-side on a display for 2,000 ms. In the GAIN condition, the choice was between a white square indicating a small gain (10 yen) with a short delay (1–7 s) and a yellow square indicating a large gain (40 yen) with a longer delay (6–26 s). In the LOSS condition, participants chose between a small loss (−10 yen) with a short delay and a large loss (−40 yen) with a longer delay. The participant indicated their choice by pressing a button; then, the black patches were removed from that square, one-by-one, at 400-ms intervals (this interval was defined as a ‘step’). When the square was completely exposed, the monetary payoff was displayed on the screen for 1,000 ms. After a 5,000–7,000-ms interval, the next trial began. The white and yellow squares were displayed with novel mosaic patterns. The initial number of coloured patches was randomly chosen from uniform distributions (white: 352 ± 32; yellow, 200 ± 120). The number of black patches removed at each step was also randomly drawn from a uniform distribution (5 ± 2). Each GAIN and LOSS session contained six blocks, and each block was finished when a total of 180 steps was achieved. In total, each GAIN and LOSS session lasted approximately 20 min. The order of the GAIN/LOSS conditions was counterbalanced across participants. Sixteen participants (ADHD: 7; NC: 9) performed six GAIN blocks first and then performed six LOSS blocks. Fifteen participants (ADHD: 7; NC: 8) performed six LOSS sessions first and then performed six GAIN blocks.

All participants were instructed to maximize monetary gains in the GAIN condition and to minimize monetary losses within the time limitation. Thus, at the beginning of each trial, the participants were required to choose between a sooner but smaller reward/loss (white) and a later but larger reward/loss (yellow) by comparing the number of black patches on the two squares. Because the participants were required to respond within 2,000 ms, decision-making was based on the visual impression of darkness on the white and yellow squares, rather than on an explicit count of the numbers of black patches and their division by the numbers of patches filled at each step. All participants received 7,500 yen as an initial monetary reward, with the total amount varying depending on their task performance. Rewards were disbursed later by direct deposit.

In this experiment, the duration of the task was fixed as described above. In detail, each block was finished when the sum of the duration (or steps) from choice to outcome in each trial exceeded the threshold (180 steps = 180 × 0.4 = 72 s). The participants were informed of this time limitation rule. Thus, each participant tried to maximize their gain per unit time and also tried to minimize their loss per unit time. If the delay for larger loss was too long, then the loss per unit time was quite small, which was the motivation for the participants to choose a larger-later loss over a smaller-sooner one. This experimental paradigm using time limitation can prevent participants from choosing sooner options independent of the size of the outcome based on delay aversion, that is, their motivation to finish the experiment as soon as possible.

The task presentation during the scan was controlled by Presentation software (version 14.5; Neurobehavioral Systems, Albany, CA). Visual stimuli were delivered to a translucent screen by an electromagnetic noise-shielded projector located at the foot of the bed. The participants viewed this screen through a tilted reflection mirror attached to the head coil. During the scan, participants held a response box and were instructed to use their right index finger to make a choice in each trial.

### Imaging acquisition and preprocessing

A 3.0-T scanner (Philips Achieva 3.0 T X-series, Amsterdam, the Netherlands) was used to acquire structural T1-weighted images and T2*-weighted echo planar images (repetition time = 2,000 ms; echo time = 50 ms; flip angle = 80°; matrix = 128 × 128; field of view = 192 mm × 192 mm; slice thickness = 4 mm; slice gap = 0 mm) with BOLD contrast. SPM12 (Wellcome Department of Imaging Neuroscience, Institute of Neurology, London, UK) was used for preprocessing and statistical analyses. We discarded the first six image volumes to avoid T1 equilibrium effects. The images were corrected for slice timing, realigned to the first image as a reference, spatially normalized with respect to the Montreal Neurological Institute (MNI) echo planar imaging (EPI) template, and spatially smoothed with a Gaussian kernel (8 mm, full width at half-maximum).

### Imaging data analysis

In the fMRI data analyses (individual level analysis), three event-related regressors were included in the GAIN and LOSS condition separately: stimulus display timing, reward display timing, and timing of each step (every 400 ms from the stimulus display until the reward display). To evaluate the effect of ‘delay’ and ‘gain/loss’ factors at the neural level, we focused on delay-related activity and outcome-related activity for both GAIN and LOSS outcomes as follows. We prepared two event-related regressors at the time of stimulus presentation for choosing a smaller-sooner option or a delayed option separately, and each event-related regressor included parametric modulation of the delay length for the chosen option. In total, we included six variables (regressors) for each session: (1) an event-related regressor for the timing of stimulus presentation when the participants chose the smaller-sooner option; (2) parametric modulation of regressor 1 with delay length; (3) an event-related regressor for the timing of stimulus presentation when the participants chose the larger-later option; (4) parametric modulation of regressor 3 with delay length; (5) an event-related regressor for the timing of each step; and (6) an event-related regressor for the timing of reward presentation.

We excluded the trials in which participants failed to press the button within the effective duration (2 s) from all regressors. All explanatory variables were convolved with a canonical hemodynamic response function and entered into a general linear model. We created images of parameter estimates for the event-related activity at the timing of stimulus displays as the interest contrasts for each participant in each condition.

In the group-level analysis, we employed two types of design. First, to evaluate group differences, we simply performed a group comparison with a two-sample t-test in the GAIN and LOSS conditions separately. Second, to evaluate an interactive effect between condition (GAIN/LOSS) and group (ADHD/NC), we employed a factorial design, in which size sensitivity or delay correlation was set as observation data, and a design matrix corresponding to factorial analysis with the condition (GAIN/LOSS) and group (ADHD/NC) as a factor was created. For visualization, we show a brain activation map with a relatively moderate threshold in Figs 3a and 4a (P < 0.05).

### ROI analysis and statistical analysis

To precisely evaluate the relationship between condition and group in the activation representing size sensitivity, we performed ROI analyses in the caudate and amygdala. In the ROI analysis of the caudate, we used an anatomically defined ROI of the caudate that included the results of our present work and our previous study^10^. We focused on the caudate because the most frequently replicated findings in neuroimaging studies in ADHD patients are structural and functional abnormalities in the caudate^70,71^ and a recent meta-analysis revealed that not only the nucleus accumbens, but also the caudate are involved in delay discounting^54^. We defined the left and right head of the caudate [left caudate head: coordinates of centre of mass = (−8.86, 13.2, 1.12), volume = 1,976 mm^3^; right caudate head: coordinates of centre of mass = (8.95, 13.1, 1.28), volume = 1,944 mm^3^] by the PickAtlas (http://fmri.wfubmc.edu/software/pickatlas). In the ROI analysis of the amygdala, we used a functionally defined ROI with a threshold of *P* < 0.05 uncorrected [coordinates of centre of mass = (27.7, −7.91, −25.9), volume = 528 mm^3^]. In both ROI analyses, we again used a general linear model with the averaged BOLD signal of all voxels within ROIs at the single-subject level and computed the value of the parameter estimates for ‘size sensitivity’ and ‘delay correlation’ averaged across participants for each GAIN and LOSS condition: ‘size sensitivity’ was defined as the subtraction of event-related activity at the timing of stimulus display for choosing smaller-sooner options from event-related activity at the timing of stimulus display for choosing delayed options, and ‘delay correlation’ was defined as a parametric modulation of delay length. We used the MarsBaR toolbox for ROI analysis. In the statistical comparisons of behavioural and ROI data analyses, we used a 2 × 2 mixed ANOVA with condition (GAIN/LOSS) as the within-subject factor and group (NC/ADHD) as the between-subject factor and also used multiple comparison analysis with the Tukey–Kramer method after ANOVA.

## Electronic supplementary material

Supplementary Information
