# Supplementary material for: Preliminary evidence of altered neural response during intertemporal choice of losses in adult attention-deficit hyperactivity disorder
Source: Sci Rep. 2018 Apr 30;8:6703. doi: 10.1038/s41598-018-24944-5 (PMC5928218; doi:10.1038/s41598-018-24944-5)
Supplement: Supplementary file 1 — Supplementary Information [file 41598_2018_24944_MOESM1_ESM.pdf]

Supplementary information

Preliminary evidence of altered neural response during intertemporal choice of losses in adult attention-deficit hyperactivity disorder

Saori C. Tanaka[1]*, Noriaki Yahata[2,4,5], Ayako Todokoro[3], Yuki Kawakubo[3], Yukiko Kano[3], Yukika Nishimura[2], Ayaka Ishii-Takahashi[3], Fumio Ohtake[6], and Kiyoto Kasai[2,7]

[1]ATR Brain Information Communication Research Laboratory Group, 2-2-2 Hikaridai, Seika-cho, Soraku-gun, Kyoto 619-0288, Japan

[2]Department of Neuropsychiatry, Graduate School of Medicine, The University of Tokyo, 7-3-1 Hongo, Bunkyo-ku, Tokyo 113-8655, Japan

[3]Department of Child Neuropsychiatry, Graduate School of Medicine, The University of Tokyo, 7-3-1 Hongo, Bunkyo-ku, Tokyo 113-8655, Japan

[4]Global Center of Excellence Program 'Comprehensive Center of Education and Research for Chemical Biology of the Diseases', The University of Tokyo, 7-3-1 Hongo, Bunkyo-ku, Tokyo 113-8655, Japan

[5]Department of Molecular Imaging and Theranostics, National Institute of Radiological Sciences, National Institutes for Quantum and Radiological Science and Technology, 4-9-1 Anagawa, Inage, Chiba 263-8555, Japan

[6]Graduate School of Economics, Osaka University, 1-7 Machikaneyamacho, Toyonaka, Osaka 560-0043, Japan

[7]The International Research Center for Neurointelligence (WPI-IRCN) at The University of Tokyo Institutes for Advanced Study (UTIAS), 7-3-1 Hongo, Bunkyo-ku, Tokyo 113-8655, Japan

*Corresponding author: xsaori@atr.jp

Supplementary Table S1

Areas showing a significant difference between groups (ADHD/NC) in size sensitivity activation in the LOSS condition (two-sample t-test).

Brain area	x	y	z	Cluster size	T-value
Fusiform gyrus (BA 37)	38	–56	–22	33	3.53
Caudate	8	6	12	40	3.39*
Precuneus (BA 7)	20	–72	54	43	3.32
Caudate	–8	2	8	34	3.19*

Peak-level threshold at $P < 0.005$ (uncorrected) and cluster-level threshold at 20 voxels.

*$P < 0.05$ (small-volume corrected) with a caudate mask.

Coordinates are in MNI space.

Supplementary Table S2

Areas showing a significant interaction between conditions (GAIN/LOSS) and groups (ADHD/NC) in size sensitivity activation (factorial design).

Brain area	x	y	z	Cluster size	T-value
Superior temporal gyrus (BA 38)	38	12	–36	52	4.36
Amygdala	24	–8	–26	23	4.05*
Superior temporal gyrus (BA 38)	–40	16	–30	28	3.69

Peak-level threshold at $P < 0.001$ (uncorrected) and cluster-level threshold at 20 voxels.

*$P < 0.05$ (small-volume corrected) with an amygdala mask.

Coordinates are in MNI space.

Supplementary Figure S1

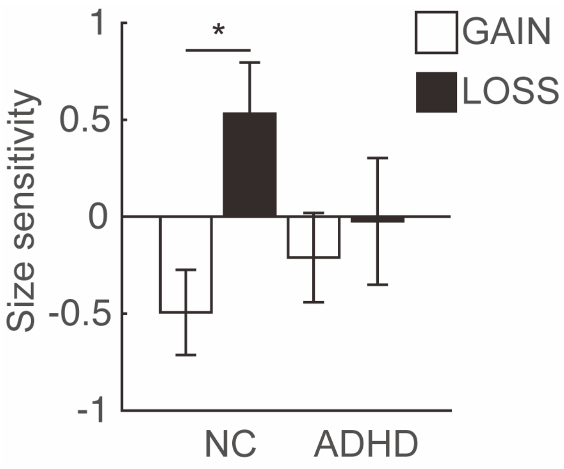

Blood-oxygen-level dependent signal for size sensitivity in the GAIN (white bars) and LOSS (grey bars) conditions in the anatomically defined region of interest of the left and right nucleus accumbens (*$P < 0.05$, multiple comparison with the Tukey–Kramer method). Error bars denote ± 1 standard error of the mean (n = 17 for NCs, n = 14 for ADHD).
